# Effects of Cocoa Antioxidants in Type 2 Diabetes Mellitus

**DOI:** 10.3390/antiox6040084

**Published:** 2017-10-31

**Authors:** Sonia Ramos, María Angeles Martín, Luis Goya

**Affiliations:** 1 Department of Metabolism and Nutrition. Institute of Food Science and Technology and Nutrition (ICTAN-CSIC). José Antonio Novais 10. Ciudad Universitaria, 28040 Madrid, Spain; s.ramos@ictan.csic.es (S.R.); amartina@ictan.csic.es (M.A.M.); 2 Spanish Biomedical Research Centre in Diabetes and Associated Metabolic Disorders (CIBERDEM), Instituto de Salud Carlos III (ISCIII), 28040 Madrid, Spain

**Keywords:** beta cell, cocoa flavonoids, flavanols, hyperglycaemia, insulin resistance, procyanidins

## Abstract

Type 2 Diabetes mellitus (T2D) is the most common form of diabetes and one of the most common chronic diseases. Control of hyperglycaemia by hypoglycaemic drugs is insufficient in for patients and nutritional approaches are currently being explored. Natural dietary compounds such as flavonoids, abundant in fruits and vegetables, have received broad attention because of their potential capacity to act as anti-diabetic agents. Especially cocoa flavonoids have been proved to ameliorate important hallmarks of T2D. In this review, an update of the most relevant reports published during the last decade in cell culture, animal models and human studies is presented. Most results support an anti-diabetic effect of cocoa flavonoids by enhancing insulin secretion, improving insulin sensitivity in peripheral tissues, exerting a lipid-lowering effect and preventing the oxidative and inflammatory damages associated to the disease. While it could be suggested that daily consumption of flavanols from cocoa or dark chocolate would constitute a potential preventive tool useful for the nutritional management of T2D, this recommendation should be cautious since most of commercially available soluble cocoa products or chocolates contain low amount of flavanols and are rich in sugar and calories that may aggravate glycaemic control in T2D patients.

## 1. Introduction

Type 2 Diabetes mellitus (T2D) is the most common form of diabetes and one of the most common chronic diseases, and its prevalence is raising worldwide [1]. T2D is characterized by a sustained hyperglycaemia due to the persistent damage in insulin secretion by pancreatic β-cell dysfunction and by insulin resistance at the peripheral tissues [1]. However, administration of glucose-lowering medications is insufficient to maintain glycaemic control in many patients and changes in life-style, such as physical exercise and nutrition, both with lowest adverse side effects, are presumed to be the most promising approaches to prevent or delay the onset of T2D. Accordingly identification of dietary components as potential antidiabetic agents has become an essential subject in the current research. Flavonoids, natural dietary compounds abundant in fruits and vegetables, have raised attention because of their prospective capacity to act as anti-diabetic agents with very few adverse side effects [1]. In particular, cocoa and its flavanols, a subfamily of flavonoids, have been proved to ameliorate important hallmarks of T2D [1]. In this review, an update of the most relevant reports published on this topic during the last decade in cell culture, animal models and human studies is presented.

## 2. Studies in Cultured Cells

Cultured cells constitute a useful tool to study the poorly understood anti-diabetic mechanism by which cocoa flavanols act in vivo. By using several cultured cell lines, very appealing and encouraging results have been reported within the last five years (Table 1).

Cocoa flavanols may improve glucose homeostasis by slowing carbohydrate digestion and absorption in the gut. Indeed, Gu et al. [2] have shown that cocoa extracts and procyanidins dose-dependently inhibited pancreatic α-amylase (PA), pancreatic lipase (PL), and secreted phospholipase A2 (PLA2). These inhibitory activities were related to the polyphenolic content in cocoa extracts and the degree of polymerization of cocoa procyanidins, showing an inverse correlation between the inhibition and degree of polymerization (Table 1). Similarly, Yamashita et al. [3] found that a cocoa liquor procyanidin extract (CLPr) (1–10 µg/mL) inhibited α-glucosidase activity in the muscle L6 myotube cells. In addition, cocoa polyphenols may also inhibit certain glucose transporters. Thus, the same CLPr increased glucose uptake in a dose-dependent manner and stimulated GLUT-4 translocation in skeletal muscle L6 cells, whereas levels of GLUT-1 and -4 remained unchanged in the plasma membrane [3] (Table 1).

Deterioration of functional β-cell mass is observed during T2D, and this critically affects to the maintenance of normoglycemia [1]. There are various studies that suggest that cocoa polyphenols may protect β-cells against death-inducing damaging factors, enhance glucose stimulated insulin secretion and induce β-cell replication. In this regard, a monomeric catechin-rich cocoa flavanol fraction enhanced glucose-stimulated insulin secretion, while cells cultured with total cocoa extract or with oligomeric or polymeric procyanidin-rich fractions did not show any effect demonstrated no improvement (all substances were used at concentrations ranged 0.75–25 µg/mL) [4] (Table 1). The increased glucose-stimulated insulin secretion in the presence of the monomeric catechin-rich fraction was associated with enhanced mitochondrial respiration (upregulation of mitochondrial complex III, IV and V components and increased cellular ATP production), suggesting improvements in β-cell fuel utilization. Additionally, the monomer-rich fraction improved cellular redox state, as increased reduced glutathione (GSH) levels and nuclear factor, erythroid 2 like 2 (Nrf2) in the nucleus and its target genes that are essential for increasing mitochondrial function [nrf1 and GA binding protein transcription factor alpha subunit (GABPA)] [4] (Table 1). Similarly, the main cocoa flavanol epicatechin (EC) (100 nM) reverted mitochondrial function-associated and biogenesis-associated indicators such as mitochondrial transcription factor A (TFAM), sirtuin (SIRT)-1, mitofilin and peroxisome proliferator-activated receptor γ co-activator 1α (PGC-1α) in high-glucose-treated endothelial cells [5] (Table 1). Further, Martín et al. [6,7] showed that a cocoa phenolic extract (CPE) and EC protected against oxidative stress in rat insulin-secreting INS-1 cells. Pre-treatment of cells with EC (5–20 µM) prevented the prooxidant *tert*-butylhydroperoxide (t-BOOH) induced reactive oxygen species (ROS), carbonyl groups, phosphorylated jun N-terminal kinase (p-JNK) expression and cell death, and recovered insulin secretion [6]. Similarly, pre-treatment of cells with CPE significantly prevented the t-BOOH-induced ROS and carbonyl groups and returned antioxidant defences [GSH levels and glutathione peroxidase (GPx) and reductase (GR) activities] to control values [7] (Table 1). Moreover, the microbial-derived flavonoid metabolites 3,4-dihydroxyphenylacetic acid (DHPAA, 5 µM), and 3-hydroxyphenylpropionic acid (HPPA, 1 µM) derived from flavanols metabolized by gut microflora potentiated glucose-stimulated insulin secretion in INS-1E cells and in rat pancreatic islets [8]. Pre-treatment of cells with both compounds protected against beta cell dysfunction and death induced by t-BOOH through the activation of protein kinase C (PKC) and extracellular-regulated kinases (ERK) pathways [8] (Table 1). In addition, procyanidin A2 (3–300 µM) prevented the damage induced on insulin secretion by bisphenol A (100 µg/L) in mice isolated islets [9] (Table 1).

Cocoa flavanols may also modulate insulin signaling, which is impaired in T2D [1]. In this line, Cordero-Herrera et al. [10,11] have reported that a CPE and EC at physiologically relevant doses (1–10 µg/mL and 1–10 µM, respectively) enhanced the tyrosine phosphorylated and total levels of insulin receptor (IR), insulin receptor substrate (IRS)-1, and IRS-2, and activated the phosphatidylinositol-3-kinase/protein kinase B (PI3K/AKT) pathway. Both natural substances also modulated hepatic gluconeogenic and phosphoenolpyruvate carboxykinase (PEPCK) expression through AKT and AMP-activated protein kinase (AMPK), and CPE increased GLUT-2 levels [10]. Indeed, EC and CPE alleviated the hepatic insulin resistance induced on HepG2 by treating the cells with a high dose of glucose [11] (Table 1). Thus, EC and CPE decreased IRS-1 Ser636/639 phosphorylation, enhanced tyrosine phosphorylated and total levels of IR, IRS-1 and IRS-2 and activated the PI3K/AKT pathway and AMPK. In addition, EC and CPE preserved HepG2 cell functionality by restoring the levels of GLUT-2, increasing glucose uptake, maintaining glycogen synthesis and decreasing glucose production [11]. Accordingly, EC and CPE pre-treatment prevented high-glucose-induced ROS generation and protein carboxylation, as well as avoided the diminution of GSH, normalized the activity of enzymatic antioxidant defences GPx, GR, catalase (CAT) and glutathione-S-transferase (GST), and phosphorylated levels of mitogen-activated protein kinases (p-MAPK)s, and maintained Nrf2 stimulation [12]. In this regard, the presence of selective MAPK inhibitors induced changes in redox status, glucose uptake, p-(Ser)- and total IRS-1 levels that were observed in CPE-mediated protection [12] (Table 1).

Early this year, cocoa procyanidin extracts have also demonstrated an insulinomimetic effect in human primary skeletal muscle cells [13]. Indeed, a procyanidin-rich cocoa extract elicited an antidiabetic effect by stimulating glycogen synthesis and glucose uptake, independent of insulin, being more pronounced this effect with larger procyanidins. In this case, cocoa procyanidins did not appear to act via stimulation of AMPK or Ca^2+^/calmodulin-dependent protein kinase II activities [13] (Table 1). Similarly, previous studies suggested that cocoa also have insulinomimetic effects in adipose tissue. Thus, a CPE (100–200 µg/mL) did not affect the levels of IR, but inhibited the IR kinase activity by direct binding, without altering total tyrosine phosphorylation of IR or inhibiting its auto-phosphorylation in 3T3-L1 adipocytes [14]. This inhibitory effect, which resulted in reduced lipid accumulation and differentiation in preadipocytes in vitro, has been related to a suppression of ERK and AKT-mediated signaling cascades to facilitate an anti-adipogenic effect; indeed, it is thought that this is one mechanism by which cocoa flavanols may inhibit the onset of obesity [14] (Table 1). In this regard, cocoa flavanols may improve blood glucose control indirectly, by modulating lipid digestion and thus, reducing hyperlipidemia and its subsequent deleterious effects on glucose homeostasis. Accordingly, Cordero-Herrera et al. [15] have reported that EC (1–10 µM) alleviated the altered lipid values induced liver cells submitted to high glucose. The lipid-lowering effect was related to diminished fatty acid synthesis (sterol-regulatory-element-binding protein-1-c and fatty-acid synthase down-regulation), and increased fatty-acid oxidation [proliferator-activated receptor (PPAR)-α up-regulation]. These effects depended on AMPK, AKT and protein kinase C ζ (PKCζ), which phosphorylated levels returned to control values upon EC treatment, playing PKCζ a role on AKT and AMPK regulation [15] (Table 1).

Cocoa flavanols may also contribute to prevent the chronic, low-grade, inflammation of T2D [16]. EC (0.5–10 µM) attenuated the TNFα-mediated down-regulation of peroxisome PPAR-γ expression and decreased nuclear DNA binding in 3T3-L1 adipocytes [17]. EC also inhibited tumor necrosis factor (TNF)α-mediated altered transcription of protein tyrosine phosphatase 1B (PTP1B), leading both effects to an attenuation of the TNFα-mediated triggering of signaling cascades involved in insulin resistance [17] (Table 1).

## 3. Animal Studies

Animal studies offer an outstanding opportunity to assess the contribution of the physiological effects of consumption of cocoa and cocoa components in different models of diabetes. Interestingly, supplementation of experimental diets with cocoa has regularly shown high acceptation by the animals and no toxicity even in chronic studies for more than 100 weeks [18]. Within the last decade different models for experimental T2D in rats and mice have been successfully used in order to study the nutritional prevention and treatment of the disease, and supplementation of the diet with cocoa has proved one of the most effective nutritional approaches (Table 2).

Administration of a diet enriched with 10% cocoa for 9 weeks to Zucker diabetic fatty (ZDF) rats reduced hyperglycaemia, enhanced insulin sensitivity and increased β-cell mass and function [19]. In particular, cocoa intake prevented β-cell apoptosis and enhanced antioxidant defences to avoid the oxidative damage and reduce lipid and protein oxidative stress (Table 2). The same treatment ameliorated circulating and hepatic lipid profiles [15], improved insulin resistance [20] and reverted hepatic oxidative stress by enhancing the antioxidant capacity of hepatocytes in ZDF rats [21] (Table 2). The lipid-lowering effect was associated to diminished fatty acid synthesis and increased fatty-acid oxidation [15]. The decreased levels of hepatic PEPCK and increased values of GK and GLUT-2 strongly collaborate to the hypoglycaemic effect of cocoa. Moreover, increased JNK and p38 induced by insulin resistance [20] and oxidative stress [21] was also reverted by cocoa (Table 2). Likewise, administration of EC to obese diabetic mice for 15 weeks, prevented fat deposition and degeneration in hepatocytes [22] (Table 2). These findings demonstrate that a diet enriched in cocoa or its main flavanol relieves hepatic insulin resistance and oxidative stress, and improves lipid metabolism, which are critical landmarks in the development and progression of T2D.

In the same line, feeding of ZDF rats for 7 weeks with a diet supplemented with a 5% of a soluble cocoa product enriched in cocoa fiber decreased blood glucose and insulin and, thus, insulin resistance measured as homeostasis model assessment of insulin resistance (HOMA-IR) index [23] (Table 2). Likewise, administration of 8% cocoa powder for 10 to male C57BL/6J high-fat-fed obese mice resulted in an improved HOMA-IR, indicating a reduced insulin resistance [24]. In the same study, cocoa feeding also reduced plasma concentration of interleukin (IL)-6, monocyte chemoattractant protein-1 (MCP-1), and increased adiponectin, changes related to a reduced inflammation characteristic of obesity and amelioration of fatty liver disease [24]. All these effects were partly mediated through the regulation of dietary fat absorption and inhibition of macrophage infiltration in white adipose tissue (Table 2). Likewise, decreased values of blood glucose without changes in body weight and food consumption were observed feeding in diabetic obese mice fed with 0.5% and 1% cocoa liquor procyanidins for 3 weeks [25] (Table 2). Furthermore, in the same mice model, administration of a cocoa liquor procyanidin extract for 13 weeks evoked a stimulation of AMPK-α, GLUT-4 translocation and uncoupling protein (UCP)-1 and -3 expression in skeletal muscle and adipose tissues, resulting in a reduced obesity, hyperglycaemia, and insulin resistance [26] (Table 2). On the contrary, no changes in glycaemia, insulinemia levels and insulin sensitivity were found in obese–diabetic rats fed a cocoa extract enriched with polyphenol and methylxanthines [27] (Table 2).

An EC-supplemented diet for 8 weeks alleviates insulin resistance in high-fructose-fed rats [28]. High-fructose feeding deactivated key proteins of the insulin signaling pathway resulting in a compromised response to insulin in the liver and adipose tissue; administration of a diet supplemented with EC partial or totally avoided these alterations. In addition, EC administration also inhibited the activation of redox-sensitive signals, expression of pro-inflammatory cytokines and chemokines, and endoplasmic reticulum stress proteins, effects that help to attenuate insulin resistance [28] (Table 2). Very recently, Cremonini and colleagues [29] have reported that administration of a high-fat diet to male C57BL/6J obese mice for 15 weeks caused obesity and insulin resistance in C57BL/6J mice as evidenced by high fasted and fed plasma glucose and insulin levels, and impaired insulin tolerance test (ITT) and glucose tolerance test (GTT) tests. This was associated with alterations in the activation of components of the insulin-triggered signaling cascade in adipose and liver tissues. In this scenario, EC supplementation ameliorated all these parameters in the high-fat fed mice, specially improving insulin sensitivity through a downregulation of the inhibitory molecules JNK, PKC and protein tyrosine phosphatase 1B (PTP1B) (Table 2). Finally, feeding of C57BL/6J mice submitted to a high-fat-diet, with a cocoa flavanol extract or three flavanol fractions enriched with monomeric, oligomeric or polymeric procyanidins for 12 weeks exerted different effects depending on the degree of flavanol polymerization [30]. Although insulin levels were lowered by all flavanol fractions, impaired glucose tolerance, insulin resistance, weight gain, and fat mass were most effectively avoided by the oligomer-rich fraction [30] (Table 2).

Therefore, most studies carried out in experimental animal models endorse the favorable effect of cocoa and its flavanols on T2D; this effect appears to be associated both to their proved beneficial effects on vascular function and on glycaemic control through the regulation of key proteins of the insulin signaling pathway and critical biomarkers of inflammation and stress in adipose tissue and skeletal muscle [1].

## 4. Human Studies

Although most cell culture and animal studies have shown an anti-diabetic activity of cocoa and its main flavonoids, translation of the results to humans is challenging and confirmation of cocoa preventive efficacy against diabetes requires large and long-lasting controlled clinical trials. Within the last few years, a significant number of systematic reviews and meta-analyses [1,31,32,33,34,35,36,37,38,39,40] point to a positive effect of cocoa and dark chocolate on improving insulin resistance, endothelial function, blood pressure (BP) and lipid profile. A number of prospective observations in longitudinal studies and intervention clinical assays have yielded analogous conclusions and a few potential molecular and biochemical mechanisms have been proposed to explain the observed benefits of cocoa and dark chocolate for diabetics [1,33,41,42,43]. All this information has been pulled out from both epidemiological and intervention studies.

*Epidemiological evidence*: Epidemiological evidence for an association between dietary intake of flavanols (whatever the source) and the risk of type 2 diabetes is large and somehow inconsistent. Three major studies could be reported as representative examples: (1) the European Prospective Investigation into Cancer and Nutrition-InterAct (EPIC-InterAct) case-cohort study including 340,234 participants with 3.99 million person-years of follow-up in eight European countries showed that a higher intake of flavanols was associated with a significantly reduced hazard of diabetes [44]; (2) the Health, Alcohol and Psychosocial factors In Eastern Europe (HAPPIE) study with 5806 participants during 4 years also concluded that intake of flavanols was significantly associated with decreased risk of T2D [45]; however, (3) in a major epidemiological study in US men and women recruited from the Nurses’ Health Studies (NHS and NHS II) and Health Professionals Follow-Up Study (HPFS) and during a 3,645,585 person-years of follow-up, only a higher consumption of anthocyanins and anthocyanin-rich fruit was associated with a lower risk of T2D, whereas no association was observed with any of the other flavonoid subfamilies [46].

Regarding studies focusing specifically on intake of chocolate and cocoa, a recent epidemiological study suggest that long-term intake of any type of chocolate may induce an inverse relation with incident T2D in young and normal–body weight men [47]. In the same line, results from the Atherosclerosis Risk in Communities (ARIC) Cohort show that the higher the frequency of chocolate intake the lower the risk of developing diabetes; a statement valid for up to 2–6 servings (1 oz) per week of chocolate. Consuming more than 1 serving per day did not yield significantly lower relative risk [48]. Very recently, two large epidemiological studies have revealed contradictory data regarding the intake of chocolate in T2D. In a prospective study longer than 30 year, a reduced incidence of T2D was observed after a moderate chocolate intake of several times per week [49]. However, in a long-term prospective cohort study in American postmenopausal women, Greenberg et al. concluded that long-term consumption any type of chocolate was unable to decrease the risk of T2D [50]. Nevertheless, the beneficial effect of cocoa and chocolate in T2D has lately received unequivocal support from meta-analysis. Hence, cocoa flavanol ingestion significantly enhanced insulin sensitivity and ameliorated lipid profile in a recent meta-analysis of a large number of randomized controlled trials [51]. Furthermore, the latest meta-analysis of prospective studies has concluded that a moderate consumption of chocolate (up to 6 servings per week) is associated with a reduced risk of coronary heart disease, stroke, and T2D [52].

*Intervention studies with chocolate*: Very promising results regarding chocolate intake and T2D have been reported during the last decade (Table 3). Thus, BP was reduced and insulin sensitivity increased in glucose-intolerant, hypertensive subjects after 15 days of consuming dark chocolate containing 1080 mg of total polyphenols/day [53]. In this study, intake of dark chocolate enriched with cocoa polyphenols diminished BP and HOMA-IR, augmented insulin sensitivity, and improved β-cell function as compared to white chocolate (Table 3). In a longer intervention during 8 weeks in diabetic patients, Mellor and colleagues [54] showed that the consumption of a high-polyphenol chocolate containing 50 mg of EC did not evoke any change in body weight, insulin resistance, BP or glycaemic control but significantly reduced the atherosclerotic cholesterol (Table 3). A later clinical trial by Almoosawi and colleagues [55] demonstrated that a 4 weeks consumption of dark chocolate containing 500 mg polyphenols by lean and overweight females evoked a significant reduction of BP and improved blood glucose control as shown by the reduced fasting glucose and HOMA-IR (Table 3). Furthermore, a relevant improvement in lipoprotein status and a significant decrease of insulin resistance have been reported after administration of chocolate enriched in flavanols and isoflavones to statin-treated diabetic women in a large study carried out in the United Kingdom (the FLAVO study) [56,57] (Table 3). Finally and more recently, Rostami and colleagues [58] reported that administration of chocolate enriched with cocoa polyphenols decreased fasting glucose and ameliorated BP in patients with diabetes and hypertension (Table 3). But not all intervention studies with chocolate have reported positive results, since fasting plasma glucose was slightly but significantly increased in overweight men after a 4 weeks intervention with dark chocolate [59] (Table 3).

*Intervention studies with cocoa and pure flavanols:* Cocoa supplementation of the diet has proved both effective and innocuous for glycaemic control in humans (Table 3). Thus, consumption of a cocoa diet enriched with flavanols (902 mg flavanols/day) for 12 weeks by overweight and obese adults considerably improved endothelial function, decreased insulin resistance and reduced diastolic and mean arterial BP as compared to those that received a low-flavanol cocoa diet [60] (Table 3). Treatment for three months with cocoa rich in EC evoked a positive regulation of oxidative stress biomarkers in skeletal muscle of patients with heart failure and T2D [61] (Table 3). A slight but significant hypoglycaemic effect has been reported in moderately hypercholesterolemic humans after administration for 2 months of a fiber-rich cocoa product providing a daily dose of 12 g of dietary fiber and 283 mg of soluble polyphenols [62] or after administration for 4 weeks of a cocoa product providing a daily dose of 416 mg of flavanols [63] (Table 3). In agreement with these results, administration for 4 weeks of a commercialized soluble cocoa product rich in dietary fiber providing 43.8 mg flavanols daily to healthy and moderately hypercholesterolemic subjects induced a slight decrease of postprandial blood glucose [64] (Table 3). In patients with T2D, cocoa powder intake for six weeks reduced total blood cholesterol, LDL-cholesterol and biomarkers of inflammation [65], and, in an acute assay, cocoa supplementation of a high-fat breakfast raised postprandial serum HDL-cholesterol and insulin [66] (Table 3). However, some studies failed to show an effect of a cocoa diet on T2D biomarkers. In a study by Muniyappa and co-workers [67], an improved endothelial function without changes in BP or insulin sensitivity was reported after ingestion of a cocoa drink rich in flavanols (nearly 900 mg of flavanols in 150 mL twice a day) for 2 weeks in patients with hypertension (Table 3). Moreover, in a study by Balzer and colleagues [68], a substantial increase in fasting flow-mediated vascular dilation along with no changes in glycaemic control, BP and heart rate were observed in T2D patients that received a diet supplemented with cocoa with a high daily dose of 963 mg of flavanols for 30 days (Table 3). Furthermore, a short-term intake of a cocoa beverage rich in flavanols by obese adults at risk for insulin resistance reduced critical markers of oxidative stress and inflammation but did not improve glucose metabolism [69] (Table 3).

Regarding administration of pure flavanols, EC supplementation to healthy adults decreased fasting blood insulin and insulin resistance (HOMA-IR) but had no effect on fasting blood glucose, BP and arterial stiffness, nitric oxide and endothelin 1 concentration, or blood lipid profile [70] (Table 3). Overall, most of the above studies support the notion that regular intake of foods rich in cocoa or cocoa flavanols could endorse a dietary strategy to appease insulin resistance. Accordingly, EC has been very recently suggested as adjuvant of metformin in the therapy for T2D patients [71].

*Cocoa and body weight*: A very exciting outcome in recent human intervention studies with cocoa is that cocoa supplementation of diets did not evoke any increase in body weight or other anthropometric changes [72,73,74]. Thus, administration of diets supplemented with up to 12.5% of cocoa powder has unequivocally shown anti-obesity effects in rats [19,20,21,24,75,76,77]. In addition, despite the fact that cocoa products commercially available are frequently high-caloric foodstuffs, they have been reported to have a similar effect in humans [72,73,74,78]. However, this anti-obesity effect of cocoa and its derivatives in humans has lately been challenged; i.e., in a prospective cohort study, Greenberg and co-workers [79] have reported a dose-response greater prospective weight gain over time after a regular chocolate consumption. In fact, the highest weight gain was reported in volunteers with the largest frequency of chocolate ingestion, which could be partially related to diminished satiety prompted by the habitual intake of chocolate. It is worth remarking that no differences among different types of chocolate (white, milk and plain) were considered in the previous work [79]. Another study by the same group in the Women’s Health Initiative cohort, reported that a greater ingestion of chocolate-candy, usually milk chocolate, was associated to a higher weight gain during a 3-year study period with postmenopausal women [80]. It ought to be stated that habitual consumption of dark chocolate, rather than milk chocolate, will prompt long-term cardiovascular benefits with a minor risk of weight gain [76]. In addition, a stronger sense of satiety and reduced need for energy intake can be more easily achieved after consumption of dark chocolate rather than milk chocolate [81], but a recent study by Esser and colleagues [59] has shown that increased flavanol content in chocolate does affect taste and has a negative effect on the motivation to eat chocolate.

In summary, most of the data above suggest that the beneficial effect of cocoa and its flavanols on T2D seems to be associated to their substantiated favorable effects on vascular function and on glycaemic control mediated through the regulation of main proteins involved in the insulin signalling pathway, as well as in the processes of inflammation and oxidative stress. The precise biochemical and molecular mechanisms have recently been reviewed [1,43]. Additionally, the European Food Safety Authority (EFSA) sanctions that cocoa flavanols help maintain normal blood pressure [82] and endothelium-dependent vasodilation [83], and, in the context of a healthy diet, the claimed effect can be obtained with a daily consumption of 200 mg of cocoa flavanols. This amount of flavanols can be acquired with the intake of 100 g of most cocoa soluble products or 40 g of any >70% cocoa chocolate in the market.

Finally, some gaps in the research to delineate the anti-diabetic effect of a cocoa intake have been detected in the literature; thus, the role of three compounds should be promptly investigated, should be investigated: (i) host and microbiota-derived flavanol metabolites; (ii) cocoa fiber and (iii) theobromine. All three have shown promising beneficial effects on cardiovascular health and body weight control and they should be considered in future controlled clinical trials.

## 5. Conclusions

Most studies within the last decade support a substantial role for cocoa and its flavanols in the nutritional prevention of T2D. Cocoa flavanols act by (a) regulating carbohydrate absorption in the gut; (b) protecting β-pancreatic cells function and enhancing insulin secretion; (c) improving insulin sensitivity in peripheral tissues such as liver, adipose tissue and skeletal muscle through regulation of glucose transporters and main proteins of the insulin signalling pathway; (d) exerting a lipid-lowering effect and; (e) preventing the exacerbated oxidative stress and inflammation characteristics of the disease. All these effects contribute to improve the insulin sensitivity and to maintain normoglycaemia, and thus, to avert and/or significantly delay the onset of T2D and development of its complications. Consequently, a moderate daily consumption of flavanols from cocoa or dark chocolate, along with an everyday intake of other dietary flavonoids, could be a valuable recommendation for the nutritional management of this disease. However, it is worth remembering that most of commercially available soluble cocoa products or chocolates contain low amount of flavanols and are rich in sugar and calories that may aggravate glycaemic control in T2D patients. Hence, recommendation of consumption of chocolate or other cocoa derivatives to this population still requires further research, especially extensive well-designed human epidemiological and intervention studies, to delineate the amount of cocoa and variety of its products that might be beneficial to prevent, delay or contribute to the treatment of T2D.

## Figures and Tables

**Table 1:** Anti-diabetic effects of cocoa flavanols in cultured cells ^a^.

Effects Related to an Anti-Diabetic Action	Cell	Cocoa Flavanol	Treatment	Reference
**Glucose uptake**				
↑ glucose uptake, ↑ GLUT-4 translocation; =GLUT-2, =GLUT-1	L6 (skeletal muscle)	Cocoa liquor procyandin extract	0.05–10 µg/mL, 15 min	[3]
**Insulin signaling**				
↑ insulin secretion, ↑ mitochondrial complex III-V, ↑ ATP, ↑ GSH, ↑ Nrf2, ↑ Nrf1, ↑ GABPA	INS-1E (pancreas)	Cocoa extract or oligomeric or polymeric-rich fraction	0.75–25 µg/mL, 24 h	[4]
↑ TFAM, ↑ SIRT-1, ↑ mitofilin, ↑ PGC-1α	HCAEC (endothelia)	EC	100 nM, 10 min or 48 h	[5]
↓ ROS, ↓ carbonyls, ↓, p-JNK, ↓ cell death, ↑ insulin secretion	INS-1E (pancreas)	EC	5–20 µM, 20 h	[6]
↓ ROS, ↓ carbonyls, ↑ GSH, ↑ GPx, ↑ GR	INS-1E (pancreas)	Cocoa phenolic extract	5–20 µg/mL, 20 h	[7]
↑ Insulin secretion, ↑ β-cell survival	INS-1E (pancreas)	3,4-dihydroxyphenylacetic acid (DHPAA), and 3-hydroxyphenylpropionic acid (HPPA)	5 µM DHPAA, 20 h; 1 µM HPPA, 20 h	[8]
↑ Insulin secretion	Mice isolated islets (pancreas)	Procyanindin A2	3–300 µM, 48 h	[9]
↑ p(Tyr)-IR, ↑ IR, ↑ p(Tyr)IRS-1, ↑ IRS-1, ↑ p(Tyr)IRS-2, ↑ IRS-2, ↑ p-AKT, ↑ p-GSK-3, ↑ p-AMPK, ↑ GLUT-2; ↓ p-GS, ↓ PEPCK, ↓ glucose production	HepG2	Cocoa phenolic extract	1–10 µg/mL, 24 h	[10]
↑ p(Tyr)-IR, ↑ IR, ↑ p(Tyr)IRS-1, ↑ IRS-1, ↑ p(Tyr)IRS-2, ↑ IRS-2, ↑ p-AKT, ↑ p-GSK-3, ↑ p-AMPK, ↓ p-GS, ↓ PEPCK, ↓ glucose production; =GLUT-2	HepG2	Epicatechin	1–10 µM, 24 h	[10]
↑ p(Tyr)-IR, ↑ IR, ↑ p(Tyr)IRS-1, ↑ IRS-1, ↑ p(Tyr)IRS-2, ↑ IRS-2, ↑ p-AKT, ↑ p-GSK-3, ↑ p-AMPK, ↑ glucose uptake; ↓ p(Ser)-IRS-1; ↓ p-GS, ↓ PEPCK, ↓ glucose production; =GLUT-2, =glycogen content	HepG2 (insulin-resistant cells)	Cocoa phenolic extract	1–10 µg/mL, 24 h	[11]
↑ p(Tyr)-IR, ↑ IR, ↑ p(Tyr)IRS-1, ↑ IRS-1, ↑ p(Tyr)IRS-2, ↑ IRS-2, ↑ p-AKT, ↑ p-GSK-3, ↑ p-AMPK, ↑ glucose uptake; ↓ p(Ser)-IRS-1; ↓ p-GS, ↓ PEPCK, ↓ glucose production; =GLUT-2, =glycogen content	HepG2 (insulin-resistant cells)	EC	1–10 µM, 24 h	[11]
↓ ROS, ↓ carbonyls, ↑ GSH, ↑ GPx, ↑ GR, ↑ catalase, ↑ GST, ↓ p-ERK, ↓ p-JNK, ↓ p-p38, ↑ Nrf2	HepG2 (insulin-resistant cells)	Cocoa phenolic extract	1–10 µg/mL, 24 h	[12]
↓ ROS, ↓ carbonyls, ↑ GSH, ↑ GPx, ↑ GR, ↑ catalase, ↑ GST, ↓ p-ERK, ↓ p-JNK, ↓ p-p38, ↑ Nrf2	HepG2 (insulin-resistant cells)	EC	1–10 µM, 24 h	[12]
↑ glycogen synthesis, ↑ glucose uptake	Human primary skeletal muscle cells	Procyanidin-rich cocoa extract	10 and 25 µM, 2 h	[13]
↓ p-ERK, ↓ p-AKT; =IR	3T3-L1 (adipocyte)	Cocoa polyphenols	100–200 µg/mL, 4 h	[14]
↓ SREBP-1c, ↓ FAS, ↑ PPAR-α, ↓ PKCζ	HepG2 (insulin-resistant cells)	EC	1–10 µM, 24 h	[15]
↓ PPARγ, ↓ PTP1B	3T3-L1 (adipocyte)	EC	0.5–10 µM, 4 h	[17]

^a^ The arrow indicates an increase (↑) or decrease (↓) in the levels or activity of the different parameters analyzed. “=” symbol indicates no changes in the parameter.

**Table 2:** Anti-diabetic effects of cocoa and cocoa flavanols in animal studies ^a^.

Effects Related to an Anti-Diabetic Action	Animal Model	Treatment	Duration	Reference
↓ Glucose, ↓ insulin, ↓ HOMA-IR, ↓ TG, ↓ LDL-Cho. ↑ HDL-Cho, ↓ NEFA	Zucker diabetic fatty (ZDF) rats	10% cocoa powder	9 weeks	[15]
↑ β-cell mass, ↑ Bcl-xL, ↓ Bax, ↓caspase-3 activity, ↑ GPx, ↑ GR, ↓ TBARS, ↓ carbonyl groups	Zucker diabetic fatty (ZDF) rats (Pancreas)	10% cocoa powder	9 weeks	[19]
↓ p-(Ser)-IRS-1, =IR, =IRS-1, =IRS-2, ↑ p-GSK3, ↓ p-GS, ↓ PEPCK, ↑ GK, ↑ GLUT-2, =p-ERK, ↓ p-JNK, ↓ p-p38 ↓ ROS, ↓ carbonyl groups, =GSH, =GPx, =GR, =CAT, ↑ SOD, ↓ GST, ↓ HO-1, ↓ p-Nrf2, ↓ Nrf2, ↓ p65-NFĸB	Zucker diabetic fatty (ZDF) rats (Liver)	10% cocoa powder	9 weeks	[20,21]
↓ fat deposition, ↑ p-AMPK	Obese–diabetic (*db-db*) mice (Liver)	0.25% EC	15 weeks	[22]
↓ Glucose, ↓ insulin, ↓ HOMA-IR	Obese Zucker fatty (ZF) rats	5% soluble cocoa fiber	7 weeks	[23]
= Glucose, ↓ insulin ↓ HOMA-IR, ↓ IL-6 ↑ adiponectin, ↓ MCP-1	High-fat-fed obese C57BL/6J mice	8% cocoa powder	10 weeks	[24]
↓ Glucose, ↓ fructosamine	High-fat-fed obese C57BL/6J mice (Adipose tissue and skeletal muscle)	0.5% and 1% cacao liquor proanthocyanidins	3 weeks	[25]
↑ p-AMPKα, ↑ GLUT-4, ↑ UCP-1,3	High-fat-fed obese C57BL/6J mice	0.5% and 0.2% cacao liquor procyanidin extract	13 weeks	[26]
=Glucose, =insulin, =HOMA-IR	Obese-diabetic (*ob-db*) rats	600 mg cocoa polyphenols/Kg body weight/day	4 weeks	[27]
↑ p-IR, ↑ p-IRS-1, ↑ ERK, ↑AKT, ↓ JNK, ↓ PKC, ↑ PTP1B, ↓ p-IKβ, ↓ IKK, ↓ p-p65-NFĸB, ↓ TNFα, ↓ MCP1, ↓ p-PERK, ↓ p-IRE1α, ↓ sXBP-1, =p-eIF2α, =ATF6, ↓ NADPH oxidase ↓ Glucose, ↓ insulin, ↓ HOMA-IR	High-fructose (HFr)-fed rats (Liver and adipose tissue)	20 mg EC/Kg body weight/day	8 weeks	[28]
↑ p-IR, ↑ p-IRS-1, ↑ ERK, ↑AKT ↓ JNK, ↓ PKC, ↓ IKK, ↑ PTP1B	High-fat-fed obese C57BL/6J mice (Liver and adipose tissue)	20 mg EC/Kg body weight/day	15 weeks	[29]
↓ Glucose, ↓ insulin, ↓ ITT	High-fat-fed obese C57BL/6J mice	25 mg oligomeric procyanidins/Kg body weight/day	12 weeks	[30]

^a^ The arrow indicates an increase (↑) or decrease (↓) in the levels or activity of the different parameters analyzed. “=” symbol designates unchanged parameters.

**Table 3:** Anti-diabetic effects of cocoa and chocolate intake in humans ^a^.

Effects Related to an Anti-Diabetic Action	Design	Population	Size	Duration (Days)	Dose (Day)	Reference
↓ HOMA-IR, ↑ QUICKI, ↑ ISI, ↑FMD, ↓BP, ↓LDL-Cho, =HDL-Cho	Randomized crossover	Hypertensive, glucose intolerant	38	15	1080 mg polyphenols	[53]
= HOMA-IR, =BP, =LDL-Cho, ↑HDL-Cho, =Glucose, =Insulin, =HbA1c	Randomized crossover	Diabetic	24	56	50 mg epicatechin	[54]
↓ HOMA-IR, ↓ BP, =Insulin, ↓ Glucose	Randomized crossover	Overweight/obese females	42	28	500 mg polyphenols	[55]
↓ HOMA-IR, =BP, ↓ LDL-Cho, =HDL-Cho, =Glucose, ↓ Insulin, =HbA1c	Randomized, placebo controlled	Diabetic	93	365	850 mg flavanols	[56,57]
↓ HbA1c, ↓ Glucose, =BP	Randomized, placebo controlled	Diabetic	60	56	450 mg flavonoids	[58]
↑ Glucose	Randomized crossover	Overweight men	44	28	1078 mg flavanols	[59]
↓ IR, ↓ BP	Randomized, controlled	Overweight/obese Volunteers	49	84	902 mg flavanols	[60]
↑ GSH, ↑ SOD, ↑ Catalase, ↓ nitrotyrosilation and carbonylation of proteins	Open label protocol	Diabetic	5	90	100 mg epicatechin	[61]
↓ Glycaemia, ↓ BP, ↓ MDA, ↑ HDL-Cho	Randomized, controlled, crossover, free-living	Moderately hypercholesterolaemic	21	60	283 mg polyphenols	[62]
↓ Glycaemia, ↓ IL-1b, IL-10, =VCAM1	Randomized, controlled, crossover, free-living	Moderately hypercholesterolaemic	44	28	416 mg flavanols	[63]
↓ Glycaemia, ↓ IL-1b, ↑ HDL-Cho	Randomized, controlled, crossover, free-living	Moderately hypercholesterolaemic	44	28	43.8 mg flavanols	[64]
↓ LDL-Cho, ↓ HDL-Cho, ↓ inflammatory markers	Randomized	Diabetic	100	42	10 g cocoa powder	[65]
↑ HDL-Cho, ↑ Ins, =LDL Cho, =TG, =Glucose, =IR, =BP	Randomized, crossover trial	Diabetic	18	Acute, 6 h	960 mg polyphenols (480 flavanols)	[66]
=BP, =glycaemic parameters	Randomized, placebo-controlled, double-blind, crossover trial	Hypertensive	20	14	Cocoa beverage (900 mg flavanols/day)	[67]
=glycaemic parameters, =BP	Randomized, double-masked fashion	Diabetic	41	30	Flavanol-rich cocoa (963 mg flavanols/day)	[68]
=Glycaemic parameters, =IL-6, =CRP	Randomized crossover design	Obese adults	20	5	Cocoa beverage (900 mg flavanols/day)	[69]
↓ IR (HOMA-IR), =Glucose, =BP	Randomized, double-blind, placebo-controlled, crossover trial	Healthy	37	28	100 mg epicatechin	[70]

^a^ The arrow indicates an increase (↑) or decrease (↓) in the levels or activity of the different parameters analyzed. “=” symbol designates unchanged parameters.
